# Liver regeneration after living donor liver transplantation (LDLT)

**DOI:** 10.1007/s00261-026-05567-1

**Published:** 2026-06-05

**Authors:** Annarita Pecchi, Francesca Camaione, Barbara Catellani, Martina Susi, Riccardo Cuoghi Costantini, Roberto D’Amico, Francesco Prampolini, Fabrizio Di Benedetto, Stefano Di Sandro, Pietro Torricelli

**Affiliations:** 1 University of Modena and Reggio Emilia, Modena, Italy; 2 Policlinico di Modena, Modena, Italy

**Keywords:** Liver regeneration rate, Regeneration velocity, LDLT, Spleen reduction rate, Body composition

## Abstract

Purpose We aim to identify possible factors affecting liver regeneration in donors, liver graft hypertrophy and spleen shrinkage in recipients. We aim also to assess the differences between donors and recipients.Methods and methodsTwenty-seven donors and 27 recipients were included. We collected anthropometric and bio-humoral parameters considered in the preoperative setting, just after the surgery, after 7 days, and after 30 days from the surgery. We calculated liver regeneration rates (RR%), regeneration velocities (RV) and spleen reduction rates (SRR%) at 2-3 months, 12 months, and 24 months. Body composition parameters were calculated.ResultsIn right lobe donors we found a mean RR of 118,70%, 146,33% and 154,88%. The mean RV was 7,94 cc/s, 0,61 cc/s, and 0,24 cc/s.For right lobe recipients the mean RR was 72,61%, 68,31%, and 79,69%. The mean RV was 9,74 cc/s, 0,19 cc/s, and 0,16 cc/s. The mean spleen reduction rate was 1,38%, 11,36%, and 10,80%.The regeneration rates were higher in donors with statistically significant differences. The recipients have a growth peak within 2-3 months, while donors at 12 months.In donors, the residual liver volume after transplantation and pre-transplant liver function are negatively correlated with regeneration. In recipients, we observed negative correlations with hematological and nutritional parameters. Liver regeneration goes parallel with the volumetric reduction of the spleen. A greater muscle mass and improved muscle quality are major stimuli to the regeneration process.ConclusionThis study emphasizes how sophisticated and versatile liver regeneration is, influenced not only by the remaining liver or graft, but also by the metabolic environment of the host.

**Supplementary Information:**

The online version contains supplementary material available at https://doi.org/10.1007/s00261-026-05567-1.

## Introduction

Liver transplantation is the only curative treatment for patients with diffuse liver diseases, liver failure, and both primary and secondary liver cancers. Due to advancements in surgical techniques, the rising incidence of tumor and non-tumor pathologies and the expansion of transplant indications, the demand for organs has grown over the years. Living donor liver transplantation (LDLT) is an innovative and complex life-saving procedure developed in the latest decades to meet the ever-growing demand for organs, given the stability of deceased donors number. The first successful LDLT was performed on children in the late 1989, using the left lateral liver segments. Later, LDLT for adults was performed with left and right lobes [1–3].

LDLT is a complex surgery compared to a cadaveric transplant with surgical risks in both recipients and donors, considering that these latter are healthy individuals; although donors face lower morbidity and mortality than recipients, there is however a mortality risk related to the size of the graft, with right hepatectomy mortality at 0.4–0.5% and left hepatectomy mortality at 0.1% [4].

At the basis of successful living donor transplants, there is the intrinsic regeneration capacity of the liver; it has been established that there is a limit beyond which the liver regenerating potential fails, which must not be exceeded to ensure a safe resection. In recipients, given the underlying disease and the presence of portal hypertension and cirrhosis, one must have a larger liver volume than the donor’s—at least 40% of the recipient standard liver volume. Whereas as little as 30% of the total liver volume is sufficient to ensure the donor's survival, who ideally is a healthy individual [1].

In this context, there is a parameter called Graft-to-Recipient Weight Ratio (GRWR), which compares the graft’s weight to the recipient’s body weight and helps determine if the graft is large enough. A GRWR of 0.8% or higher is generally considered sufficient, whereas lower values are associated with the risk of Small-for-Size Syndrome (SFSS), where smaller grafts may fail to meet metabolic demands, leading to liver failure [1].

An important goal of preoperative LDLT study is calculation of liver volumes. Liver volume assessment is most commonly performed on computed tomography (CT) or magnetic resonance (MR) imaging, but both techniques tend to overestimate the true liver volume of the resected graft. This is mainly due to inclusion in the volume of vascular structures, so a conversion factor of approximately 0.8 is generally applied to determine the real functional graft tissue volume [1].

Therefore, the most common graft used is the right lobe which represents 60% of the total liver volume [4].

The regeneration is an intrinsic capacity within normal hepatic tissue and is a finely orchestrated process that involves several interrelated extracellular and intracellular mechanisms, particularly a complex interplay of hepatomitogens and other factors that start, promote, and terminate the process [5–8].

At the core of regeneration, there are two sequential processes: hyperplasia and hypertrophy. The first a cellular division, is caused by growth factors such as interleukin-6 (IL-6), tumor necrosis factor (TNF), hepatocyte growth factor (HGF), epidermal growth factor (EGF), and fibroblast growth factor (FGF). The latter, hypertrophy, is defined by hepatocyte polyploidization, in which hepatocytes increase their DNA content without undergoing cell division, resulting in cells with multiple nuclei, which exhibit greater metabolic and synthetic capacities other than being larger [6].

Cell division is rarely observed in the adult liver as the cells are in the G0 phase of the cell cycle, so they are quiescent; after up to 70% resection or damage, cells rapidly reenter the cell cycle. Hepatocytes are the first to reenter the cell cycle followed by nonparenchymal cells (Kupffer cells and endothelial biliary cells) [6].

The main mechanism behind these regenerative processes is the hemodynamic change caused by the redistribution of portal flow to the remaining liver, which raises portal pressure and causes stress over the vascular walls (also referred to as "shear stress"). This triggers the upregulation of genes synthesizing factors that initiate the regeneration process [6].

Moreover, hemostasis not only plays a crucial role in stopping bleeding but also in regeneration. Platelets rapidly migrate to the space of Disse, where they stimulate hepatocyte proliferation by releasing VEGF, HGF, and serotonin [9].

Various studies have identified numerous other factors that positively or negatively affect the regeneration process: aging causes a progressive decline in the regenerative capacity of organs and tissues [10, 11], female sex is positively correlated with regeneration due to the role of estrogens [12, 13], the pancreas generally plays a role in liver regeneration by secreting hepatopietin A (a mitogen) and exocrine production triggers hepatocyte proliferation [14]. Moreover, diabetes and conditions that result in altered insulin secretion and IGF can impair regeneration since insulin is a significant hepatotropic agent [15], cholestasis lowers portal venous flow, lowering hepatotropic factor production [16]. At the same time, through the stimulation of Kupffer cells, elevated bile salts also promote regeneration [17]. Immunosuppression therapy and others drugs used after transplantation**,** can affect regeneration. [18]. Residual liver volume after surgery is inversely correlated with regeneration [19–22]. Higher BMI values are correlated with greater regeneration [19]. Low preoperative ALT values, meaning better liver function, favor regeneration [21]. Preservation or reconstruction of the MHV (middle hepatic vein) in the graft or the residual liver reduces venous congestion and therefore enhances regeneration [23, 24].

After liver transplantation, a reduction in spleen volume is observed, primarily driven by hemodynamic changes following the transplant, particularly the resolution of portal hypertension due to the restoration of normal hepatic architecture; as a result, as portal pressure falls, splenic congestion decreases. This drop in spleen volume is also related to a decrease in hypersplenism, and hence, platelets and other blood cells sequestration, with the return of platelet count within normal ranges [25].

In general, after an initial period of major reduction (up to 6 months), mainly due to the normalization of portal pressure, there is a slower and continuous phase of reduction that can last for many years after transplantation, reflecting the gradual regression of chronic structural changes, such as fibrosis. This is more common in severe and long-standing splenomegaly, that may persist for up to 4 years [26].

A factor affecting spleen shrinkage is the liver graft weight [27, 28], although contrary to what was expected, no differences were found in the amount of spleen reduction and the recovery of platelet count between total transplant (cadaveric transplantation) and partial transplant (LDLT) [27, 29]. As suggested by Kim et al. [27] one reason for this discrepancy is that even though cadaveric transplantation offers a larger graft than LDLT, the regeneration capacity of a liver-living graft is superior to that of a cadaveric liver. Another possible explanation is that there might be a threshold for graft weight or volume necessary to decompress portal pressure to normal ranges, beyond which no further spleen reduction would occur.

In the literature there is evidence of how conditions such as sarcopenia, a syndrome characterized by progressive and generalized loss of skeletal muscle mass and strength, and sarcopenic obesity, a condition where obesity coexists with low muscle mass and strength, negatively influence the outcome of various pathologies such as pancreatic, lung and colon cancer [30–32]; it is also reported that these conditions negatively influence the liver regeneration process in the recipient after transplantation from a cadaver or living donor. This suggests that liver regeneration is strongly related to various local and/or systemic factors such as body compositions parameters [33–35].

So the role of imaging is paramount not only for surgery planning and possible surgical complications, but also, in the evaluation of liver volumetry in the different time points of LDLT; in fact, both CT and MR allow the calculation of liver volume through dedicated software, to plan the transplant and to follow the complex regenerative process after the transplant in the donor and recipient [1, 2].

In our study we aim to identify possible factors affecting liver regeneration in donors, liver graft hypertrophy and spleen shrinkage in recipients and to assess the differences in liver regeneration between donors and recipients in the setting of living donor liver transplantation (LDLT).

## Materials and methods

This retrospective study was approved by the Ethics Committee [prot AOU n° 0020179/22 of 7/7/2022] and Informed consent has been collected.

We considered living donor liver transplantations (LDLT) carried out at Modena hepato-biliary pancreatic surgery and liver transplant unit in the period from July 2020 to November 2023.

### Donors

We collected anthropometric parameters like age, weight, height, and body mass index (BMI) and bio-humoral parameters like AST, ALT, GGT, bilirubin, platelets, and CRP preoperative, just after the surgery, at 7 days and 30 days postoperative.

All the donors underwent preoperative imaging studies, including computed tomography and magnetic resonance, to evaluate transplant eligibility. After LDLT the donors underwent also MR follow-up at 2–3 months, 12 months, and 24 months.

All the CTs were obtained using a 64-slice MDCT scanner and a standardized triphasic liver protocol with injection of intravenous iodinated contrast medium (Iohexol 300 mg I/mL, Omnipaque, GE Healthcare) at a dose of 1,5 mL/Kg of body weight and at a flow rate of 3 mL/second.

All the MRs were obtained using a 1,5 T MR scanner and liver protocol. The preoperative donors' MR examinations were performed with hepatospecific contrast agent (0,1 ml/Kg Gd-EOB-DTPA at a flow rate of 0,8 mL/second). In the follow up the MRs were performed with extracellular or hepatospecific contrast agents chosen based on the clinical indication (0,2 ml/Kg Gd-DOTA or 0,1 ml/Kg Gd-EOB-DTPA).

All the liver volumes obtained at radiological, CT and MR, imaging were manually determined by hand-tracing the surface areas of each slice (from MR and CT images), carefully excluding large vessels and major fissures. Then, through automated software calculation, we obtained liver volumes, expressed in cm^3^.

**Fig. 1:**
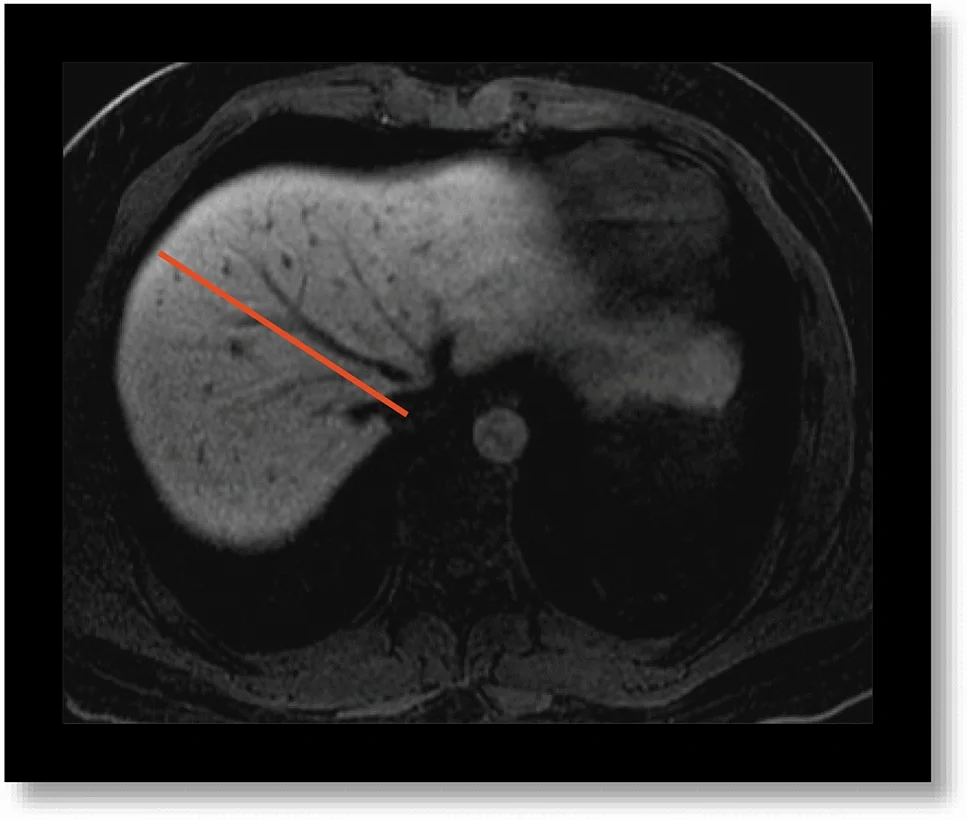
MR image with Cantile imaginary line used to define the standard section plane for right and left lobe volume calculation, drawn about 1 cm to right of the middle hepatic vein

At preoperative donors imaging we calculated:

- **Total liver volume (cm**^**3**^**)** on both portal venous phase CT images and 3D T1 hepatobiliary MR images, calculating the concordance of the values ​​obtained with the two methods.
- **The volume of the right and the left lobe (cm**^**3**^**)** on 3D T1 hepatobiliary MR images. The standard section plane used on imaging to divide right and left lobes was drawn about 1 cm to right of the middle hepatic vein along the Cantile imaginary line (Fig. 1). This line is considered the surgical resection line that divides the liver into two hemi-livers, the right and left.

We also collected the **weight back table (WBT)** in grams of the graft detected on the scale in the operating room during the transplant.

At follow-up MR imaging at 2–3 months, 12 months, and 24 months we calculated:

- **Liver remnant volume (cm**^**3**^**)** over time on the second phase or portal phase of the dynamic study in the MR with Gd-DOTA and on the hepatobiliary phase for the MR with Gd-EOB-DTPA.
- **Regeneration rates (RR%)** at different time points using the following formula:
  $$ \left[ {\left( {{\text{remnant liver volume at follow}} - {\mathrm{up}}{-}{\mathrm{FRLV}}} \right)/{\mathrm{FRLV}}} \right] \times {1}00 $$

where FRLV is the residual liver volume after resection, calculated in preoperative donor MR images.

- **Regeneration velocity (RV) (cm**^**3**^**/days)** at different time points using the formula:
  $$ {\text{difference of growth volume between time points}}/{\text{number of days}} $$

**Fig. 2:**
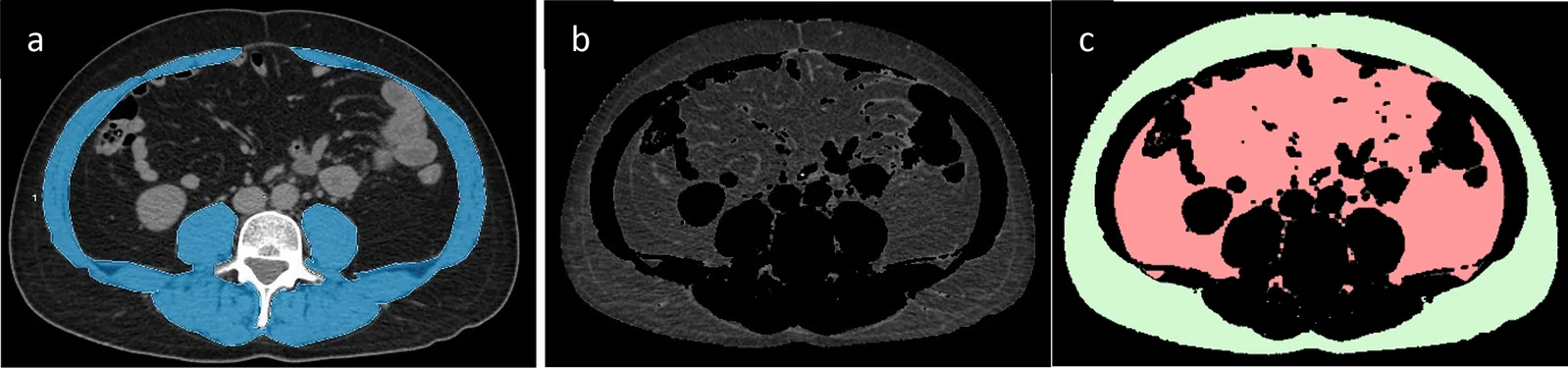
Axial CT images at the L3 level highlighting the selected muscle compartement (**a**: blue) and the adipose tissue (**b**: grey) divided into subcutaneous adipose tissue (**c**: green) and visceral adipose tissue (**c**: pink) for the calculation of body composition parameters

We also analyzed the body composition parameters muscle and fat related obtained from preoperative pre-contrast CT images (Fig. 2); in particular we selected a single CT slice at the third lumbar vertebra (L3) level where both transverse processes were clearly identifiable. A GE AW Volume Share 7 workstation, equipped with specialized software, was used; the software allows the selective visualization of both muscle, by establishing threshold values within a density range from −29 to + 150 Hounsfield Units (HU) and fat tissue, by establishing threshold values within a density range from – 180 HU to – 30 HU. Areas of interest (ROI) were manually delineated using the software tool, aligning with the designated compartments for analysis. Within these ROIs, the software automatically computed the area expressed in square centimeters and the average density value. We considered the following muscle-fat parameters:

- Psoas muscle area (PMA),
- Skeletal muscle areas (SMA) that included: psoas, erector spinae, quadratus lumborum, transversus abdominis, external and internal obliques, and rectus abdominis,
- Visceral fat areas (VFA) calculated after cutting the subcutaneous fat from the image with selective tissue adipose visceral and subcutaneous
- Subcutaneous fat areas (SFA) calculated after cutting the visceral fat from the image with selective tissue adipose visceral and subcutaneous
- Total fat area (TFA) understood as the sum of the area of ​​visceral and subcutaneous fat
- SMA, VFA, SFA and TFA were normalized for patients’ height squared obtaining the corresponding indices: skeletal muscle index (SMI), total fat index (TFI), visceral fat index (VFI), and subcutaneous fat index (SFI),
- Muscle attenuation (MA) positioning a region of interest (ROI) at the psoas muscle level,
- Subcutaneous tissue density positioning a ROI at the level of the ipsilateral subcutaneous adipose tissue,
- Intramuscular adipose content (IMAC): ratio between MA and subcutaneous tissue density,
- Visceral adiposity (VSR): visceral to subcutaneous adipose tissue area ratios (VSR) to understand abdominal adipose tissue distributions.

### Recipients

We considered the preoperative MELD score (Mayo End Stage Liver Disease) and Child–Pugh score.

Also in this group we collected the same anthropometric parameters like age, weight, height, and body mass index (BMI) and bio-humoral parameters like hemoglobin, hematocrit, platelets, AST, ALT, GGT, bilirubin, albumin, and CRP preoperative, just after the surgery, at 7 days and 30 days postoperative.

All recipients underwent preoperative CT imaging within two weeks of the transplant.

Similarly to the donor we selected post-operative CT examinations performed at 2–3 months, 12 months and 24 months follow-up times. The CT scans in the recipients were performed with the same equipment, same contrast medium and same triphasic liver protocol used in the donors.

We considered:

- **Graft volume (cm**^**3**^**)** at the time of transplant estimated indirectly from preoperative donors’ MR images.
- **Graft-to-recipient weight ratio (GRWR).** This value represents the ratio between the graft weight and the recipient's body weight. This ratio helps to evaluate if the graft is big enough to sustain metabolic, synthetic, and hemodynamic recipients’ needs and avoid the small-for-size syndrome (SFSS), with the standard cut-off considered ≥ 0,8% for good transplant outcomes.

At follow-up CT imaging at 2–3 months, 12 months, and 24 months we calculated:

- **Liver volume (cm**^**3**^**)** over time on the portal venous phase CT images.
- **Regeneration rates (RR%)** at different time points using the following formula3:
  $$ \left[ {\left( {{\text{remnant liver volume at follow}} - {\mathrm{up}}{-}{\mathrm{FRLV}}} \right)/{\mathrm{FRLV}}} \right] \times {1}00 $$

where FRLV is the liver volume after transplant, calculated in preoperative donors’ MR images.

- **Regeneration velocity (RV)** (cm^3^/days) at different time points using the formula:
  $$ {\text{difference of growth volume between time points}}/{\text{number of days}} $$

Furthermore, in the recipients we calculated spleen volume at pre operative and over time in the same follow-up CTs. The following formula was used to evaluate **spleen shrinkage** in recipients at different time points:

$$ \left[ {\left( {{\text{volume at t}}0{-}{\mathrm{follow}} - {\text{up volume}}} \right)/{\text{volume at t}}0} \right] \times {1}00 $$

The t0 volume is the spleen volume before transplantation, calculated from preoperative recipients' CT images.

We also calculated in the same way of donors the **body composition parameters** on the preoperative CT of the recipients, in particular:

- Psoas muscle area (PMA),
- Skeletal muscle area (SMA),
- Skeletal muscle index (SMI),
- Muscle HU (MA),
- HU adipose subcutaneous tissue,
- Intramuscular adipose tissue content (IMAC),
- Visceral fat area (VFA),
- Subcutaneous fat area (SFA),
- Total fat area (TFA)
- Visceral fat index (VFI)
- Subcutaneous fat index (SFI),
- Total fat index (TFI),
- Visceral adiposity (VSR).

### Statistical analysis

Data handling and analyses were performed using R software (version 4.3.2).

For categorical variables, collected data were described using absolute and relative frequencies, whereas continuous numerical variables were described using median and standard deviation (SD) or median and interquartile range (IQR).

Using simple linear regression and multiple linear regression, in each group of donors and recipients, we compared the regeneration rates and regeneration velocity at different time points with the collected parameters which are anthropometric, bio-humoral, and body composition parameters and total liver volume before hepatectomy for donors, and anthropometric, and bio-humoral parameters, total liver volume and spleen volume before transplantation, GRWR ratio, MELD, and Child–Pugh score for recipients.

Furthermore, using the same models, we compared regeneration rates and regeneration velocity between the two groups.

Linear mixed models were used to estimate the time trend of regeneration rates and regeneration velocity. These models included a random intercept term to account for repeated measurements on the same individuals. Also, an interaction term between time and the collected parameters of interest was considered to investigate how these parameters modify the relationship between time and outcome.

In each model, only subjects without missing values in the variables involved in the model were considered, as imputation of missing values was not performed.

The results obtained from regression models were reported in terms of regression coefficients (Beta, B) with their 95% confidence intervals (95% CI) and p-values. This was an exploratory analysis, and no multiple testing correction has been applied. P-values ≤ 0.05 were considered statistically significant.

## Results

From July 2020 to November 2023, 27 living donor liver transplantations (LDLT) were carried out at Modena hepatobiliary pancreatic surgery and liver transplant unit, including 23 right lobe and 4 left lobe transplants.

All donors were studied with EOB-MR and CT 2 weeks before transplantation.

All recipients were studied with CT 2 weeks before transplantation.

At this time, 24 donors (20 right hepatectomies and 4 left hepatectomies) already underwent MR at 2–3 months, 20 patients (19 right hepatectomies and 1 left hepatectomy) at 12 months, and 9 patients (all right hepatectomies) at 24 months.

On the other hand, 21 recipients (3 left lobe recipients and 18 right lobe recipients) already underwent CT at 2–3 months, 18 patients (3 left lobe recipients and 15 right lobe recipients) at 12 months, and 9 patients (all right lobe recipients) at 24 months.

### Regeneration rate

With regard to all 27 donors, the mean regeneration rates were 102.35% at 2–3 months, 140.39% at 12 months, and 154.88% at 24 months. Separating left and right hepatectomy lower values in left hepatectomies (mean regeneration rate at 2–3 months of 20.57%) and higher rates in right hepatectomies (mean regeneration rate at 2–3 months of 118.70%) are observed.

Whereas evaluating all the 27 recipients, there is a mean regeneration rate of 69.22% at 2–3 months, 66.97% at 12 months, and 79.69% at 24 months. Even in this group, there are differences, although less pronounced, between left and right recipients and in particular a mean regeneration rate at 2–3 months of 72.61% in right recipients and 48.92% in left recipients, and at 12 months a mean regeneration rate of 68.31% in right recipients and 60.24% in left recipients.

Right hepatectomy represents a greater regenerative stimulus on the residual left lobe while left hepatectomy induces a slower regeneration. Instead, in the recipients the regeneration rate is higher when the right lobe is transplanted.

### Regeneration velocity

While the mean regeneration velocity in donors was 7.19 cc at 2–3 months, 0.60 cc at 12 months, and 0.24 cc at 24 months, even in the velocity, there is little difference in left and right hepatectomy (mean velocity of 7.94 cc in right hepatectomy and 3.44 cc in left hepatectomy at 2–3 months).

The regeneration velocity in the donor is also greater in right-hepatectomy than in left-hepatectomy.

In recipients, the mean regeneration velocity was 9.39 cc (9.74 cc in right hepatectomy and 7.34 cc in left hepatectomy) at 2–3 months, 0.22 cc (0.19 in right hepatectomy and 0.40 in left hepatectomy) at 12 months and 0.16 cc at 24 months.

Therefore, considering the differences reported above, and the small number of patients who underwent left transplants, we decided to rule out these patients and analyze just the 23 donors who underwent right hepatectomies and their corresponding recipients.

### Donors

Our 23 donors included 12 females and 11 males (52.2% and 47.8%) with a mean age at the time of resection of 45 (range 23–65) and a median BMI of 24.4 kg/m^2^ (range 18.8–31.3).

### Bio-humoral parameters

Almost every preoperative bio-humoral parameters were within the normal range (Table S1). At the same time, the liver function indexes (mostly ALT and AST) increased just after the surgery (Table S2) with a progressive normalization of the values at 7 days (Table S3) and 30 days postoperative (Table S4).

### Body composition

As to the body composition parameters (Table S5), particularly muscle mass, only in one patient the values of SMA and SMI fell at the lower limit of normal ranges although with normal MA values [32, 36]. The intramuscular adipose content (IMAC), representing the fat stored within the muscle and a critical factor influencing muscle quality, was in the normal range in all patients.

For the VSR (VFA/SFA), we found slightly elevated values above the normal range in four patients [32]; in three of them, the VFA values were above 130 cm^2^, considering this value the cut-off beyond which metabolic and cardiovascular risk increases [37, 38]. The regeneration rates of these patients do not diverge much from the average regeneration.

### Volume graft

Examining the liver volumes (Table 1) calculated through imaging, the mean right lobe volume in cm3, manually calculated, was 806 (± 136), and after conversion in grams, a mean of 734 (± 124), that proves to be in line with the weight graft measured on the scale in the operating room (mean of 736 g ± 107). In our cohort, the imaging proved to be reliable in predicting the liver weight, although, the volume expressed in cm3 tends to overestimate the graft weight assessed on the back table. Using the conversion factor, the average value of the graft weight in grams is in line with the weight found at back table in the operating room.

**Table 1:** Liver volumes

Variable	N	Missing	Mean	Median	SD	Minimum	Maximum
TLV (cm3)	23	0	1289	1254	208	861	1852
RLV (cm3)	23	0	806	768	136	573	1140
RLV conver (grams)	23	0	734	699	124	521	1037
WBT (grams)	23	0	736	748	107	504	950

The table shows the mean value of TLV (total liver volume) and RLV (right liver volume) expressed in cm3 compared with RLV converted into grams and the weight of the right liver lobe detected on the scale in the operating room expressed in grams. *TLV* total liver volume, *RLV* right liver volume, *RLV conver* right liver volume converted in grams, *WBT* weight back table, *SD* Standard Deviation

### Regeneration rate (RR)

The mean percentage of volumetric recovery compared to the total liver volume before transplantation was 74.06% at 2–3 months, 87.48% at 12 months, and 94.20% at 24 months. Remarkably, all 19 patients who already underwent the 12-month follow-up exceeded 70% hypertrophy, and, although only one patient achieved hypertrophy levels exceeding 100% of the initial liver volume, all the levels of hypertrophy are sufficient to restore liver functionality.

The analysis of the regeneration rates at the established follow-up times highlighted a mean regeneration rate of 118.70% at 2–3 months, 146.33% at 12 months, and 154.88% at 24 months.

**Graph 1:**
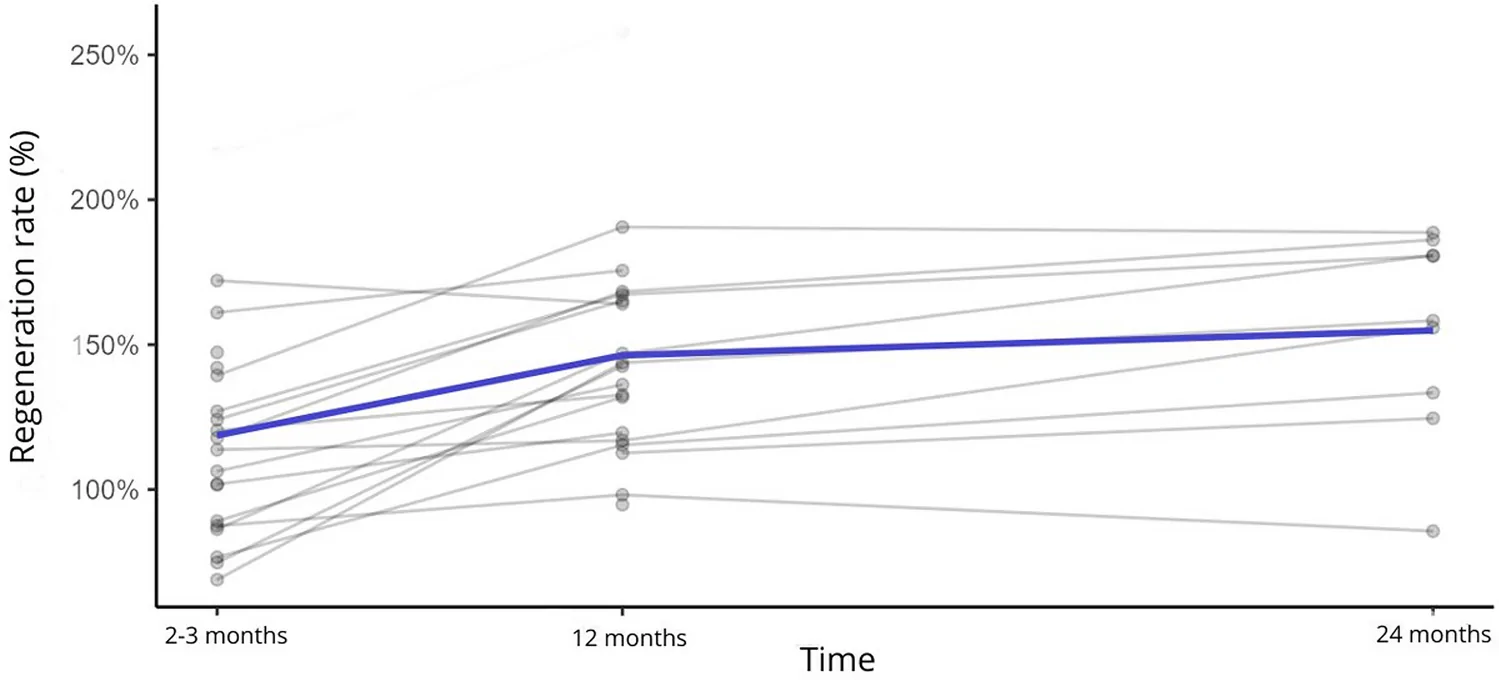
Regeneration rate (%) in donors over time

Moreover, the peak of regeneration is at 12 months (Graph 1), when the regeneration process is almost completed.

#### Statistically significant correlations of the regeneration rate

**Analyzing the variation of regeneration rates over time, we found a negative correlation with the residual liver volume just after transplantation** (p = 0.005, B = −0.19,0.95% CI:[−0.07, −0.31]), that is major regeneration for lower liver volume at time 0, and a **negative** correlation with preoperative ALT (p = 0.026, B = 0.02, 95% CI:[−0.04, −0.003]), so lower values lead to major regeneration.

While studying the single regeneration rates we found a **negative** correlation at 2–3 months with height (p = 0.006, B = −0.03, 95% CI:[−0.06, −0.01]), and a **negative** correlation with postoperative bilirubin (p = 0.049, B = −0.18,0.95%CI: [−0.36, −0.01]).

At 24 months we had a **negative** correlation with ALT and AST at 30 days (respectively p = 0.012, B = −0.02, 95%CI: [−0.01, −0.03] and p = 0.012, B = −0.01.95%CI: [−0.01, −0.004]).

### Regeneration velocity (RV)

**Graph 2:**
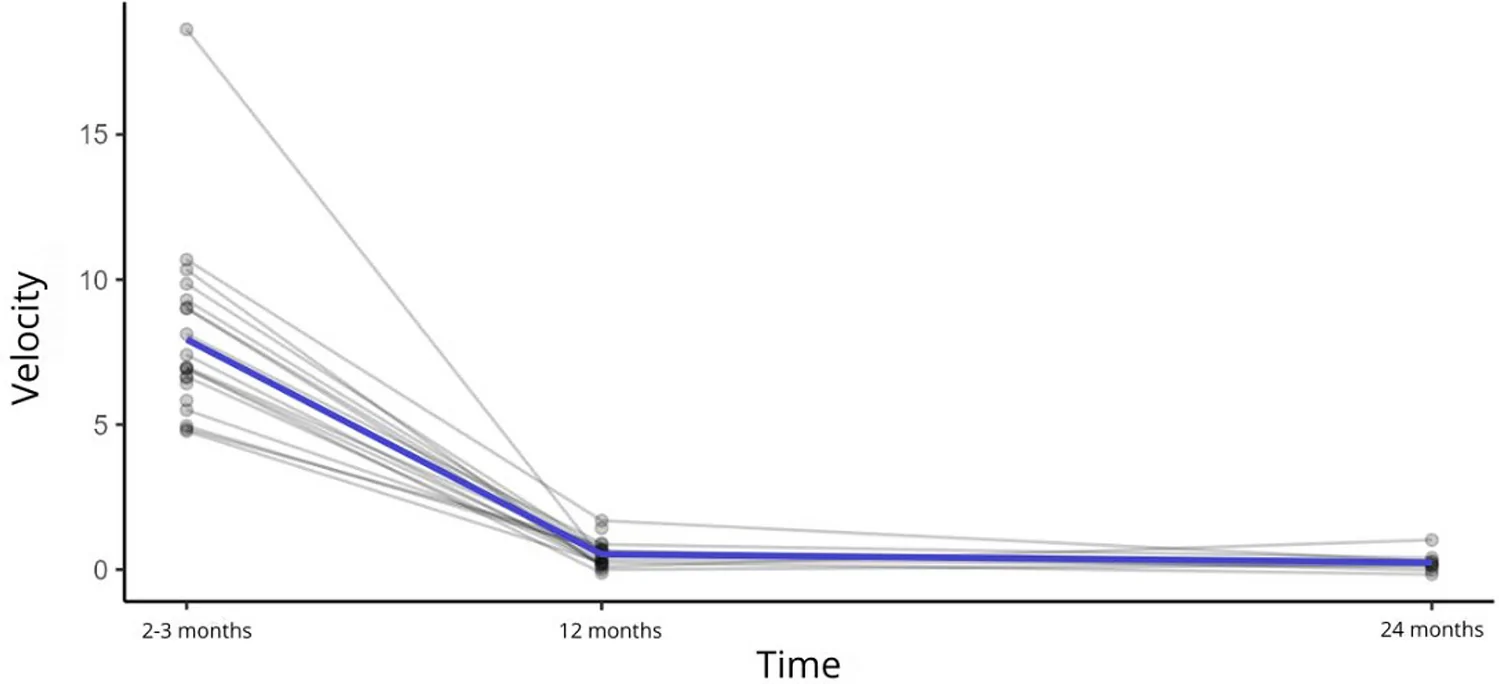
Regeneration velocity in donors over time

**The mean regeneration velocity was 7.94 cc at 2–3 months, 0.61 cc at 12 months, and 0.24 cc, with the maximum velocity peak already at 2–3 months** (Graph 2).

#### Statistically significant correlations of the regeneration velocity

The variation of velocity regeneration from 2–3 months and 12 months was **negatively** correlated with height (p = 0.026, B = −0.22, 95%CI: [−0.40, −0.04]), **positively** with CRP at 30 days (p = 0.003 B = 3.40, 95%CI: [1.29, 5.51]), and **negatively** with MA (p = 0.006, B = −0.45, 95%CI: [−0.76, −0.15]).

Evaluating just the regeneration velocity at 12 months we found a correlation with gender (p = 0.030 B = 0.45, 95%CI: [0.08, 0.82]) with a mean RV higher in males (0.73) than in females (0.35).

For body mass composition a **positive** correlation with SMA (p = 0.002, B = 0.01, 95%CI: [0.01, 0.02]), SMI (p = 0.005, B = 0.04, 95%CI: [0.01, 0.06]), HU adipose subcutaneous Tissue (p = 0.003, B = 0.03, 95%CI: [0.01, 0.05]), and VSR (p = 0.015, B = −0.46, 95%CI: [0.13, 0.79]), and a **negative** correlation with IMAC (p = 0.001, B = −4.33, 95%CI: [−6.49, −2.18]) can be noticed.

### Recipients

The 23 recipients included 8 females and 15 males (65.2% and 34.8%) with a mean age at the time of transplant of 65 (range 29–75) and a median BMI of 26.3 kg/m^2^ (range 19–34.8). This group of patients appears to be very heterogeneous, also due to the different underlying diseases that led to the indication for transplantation namely: HCC (14), cirrhosis (5), multifocal epithelioid hemangioendothelioma (1), primary sclerosing cholangitis (1), primary biliary cholangitis (1), and Lyell's syndrome (1). Two of these patients died after transplantation, both developed metastases after liver transplantation and died due to disease progression despite chemotherapy treatment.

Evaluating the bio-humeral parameters, most values are above the normal range, both preoperatively and postoperatively (Tables S6-S9).

### Body composition

As for body mass composition parameters (Table S10), compared to the donors, we observed lower values for muscle mass and higher values for adipose tissue. In particular, 7 patients out of 23 had SMI values below the cut-off points (34.4 cm^2^/m^2^ for females and 45.4 cm^2^/m^2^ for males), 5 of whom also had reduced SMA values (92.2 cm^2^ for females and 144.3 cm^2^ for males) [36]. These patients did not have a BMI within the obesity range.

For the adiposity, we found ten patients with VFA values above 130 cm^2^, four of which had BMI in the obesity range [37, 38]. Furthermore, VSR was above the optimal range in seven patients [32]. Notwithstanding these, we did not find any statistically significant correlation between RR% and body composition parameters, despite a positive correlation between RV at 2–3 months and PMA, SMA, SMI (respectively p = 0.008, B = 0.96, 95%CI:[0.34, 1.58]; p = 0.002, B = 0.15, 95%CI:[0.07, 0.24]; and p = 0.002, B = 0.55, 95%CI: [0.25, 0.85]).

### Volume graft

The mean percentage of volumetric recovery compared to the estimated volume according to the Vauthey formula [39] that predict total liver volume based on body surface area (BSA) or body weight was 84.70% at 2–3 months, 82.37% at 12 months, and 87.10% at 24 months. In recipients, notwithstanding the lower regeneration rate, 11 of the 15 patients who already underwent follow-up at 12 months reached hypertrophy of over 70% of the estimated liver volume.

### Regeneration rate (RR)

The mean regeneration rate was 72.61% at 2–3 months, 68.31% at 12 months, and 79.69% at 24 months.

**Graph 3:**
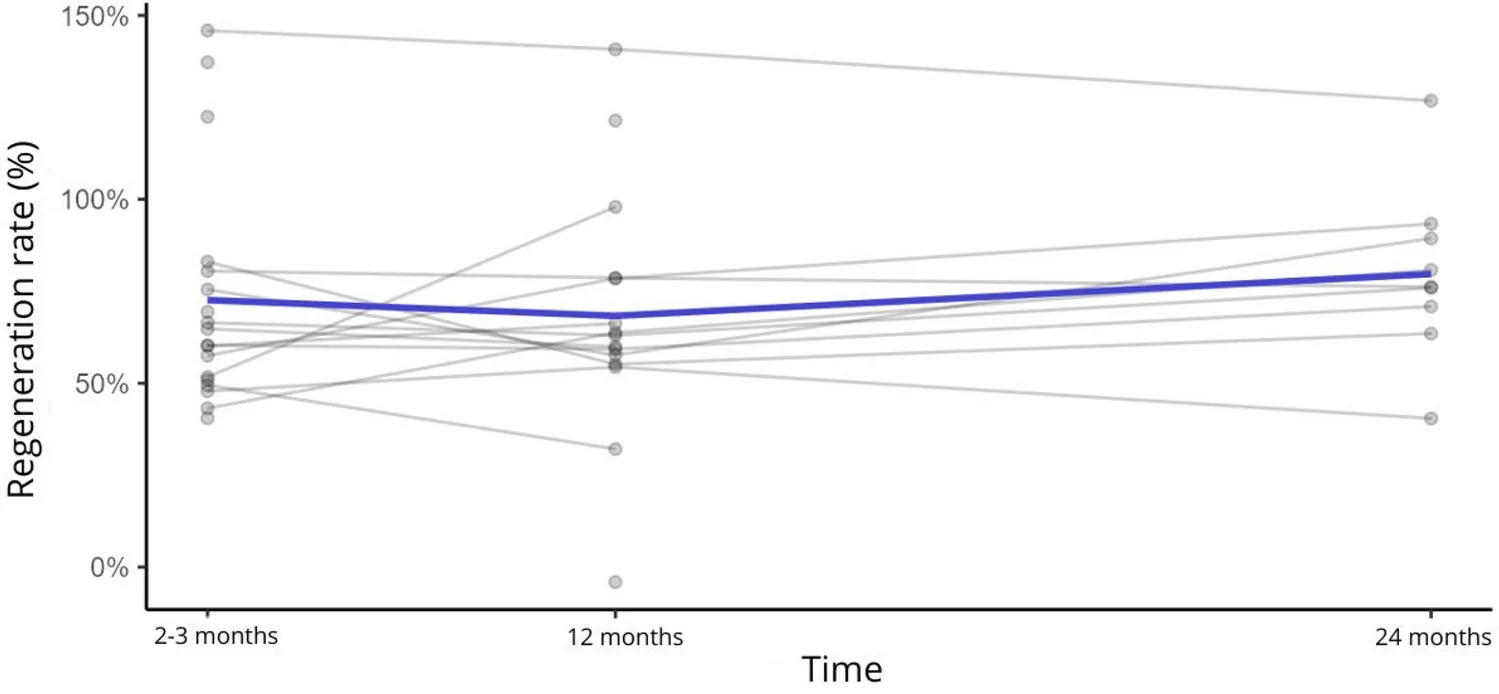
Regeneration rate (%) in recipients over time

Furthermore, unlike the donors, the peak in regeneration rate (Graph 3) was already at 2–3 months, with slightly little progression afterward.

#### Statistically significant correlations of the regeneration rate

The variation of regeneration rate over time **positively** correlated with spleen volume before transplantation (p = 0.01, [0.02, 0.10]).

Furthermore, the regeneration rate at 2–3 months was correlated with gender (p = 0.035, B = 0.31, 95%CI: [0.05, 0.58]) with a higher regeneration rate in males (mean of 84.80% in males and 53.45% in females).

We found a **negative** correlation between the RR% at 12 months and preoperative hemoglobin and hematocrit (respectively p = 0.015, B = −0.12, 95%CI:[−0.21, −0.04] and p = 0.024, B = −0.04, 95%CI:[−0.07, −0.01]), and also a **negative** correlation between the RR% at 24 months and preoperative hemoglobin and hematocrit (respectively p = 0.015, B = −0.12, 95%CI:[−0.21, −0.05] and p = 0.027, B = −0.04, 95%CI:[−0.08, −0.01]).

The RR% at 24 months was **negatively** correlated, as well, with height (p = 0.044, B = −0.02, 95%CI:[−0.03, −0.003]), and with preoperative albumin (p = 0.037, B = −0.28, 95%CI:[−0.49, −0.07]).

### Regeneration velocity (RV)

The mean regeneration velocity was 9.74 cc, 0.19 cc, and 0.16 cc, with the maximum velocity peak at 2–3 months.

#### Statistically significant correlations of the regeneration velocity

**Graph 4:**
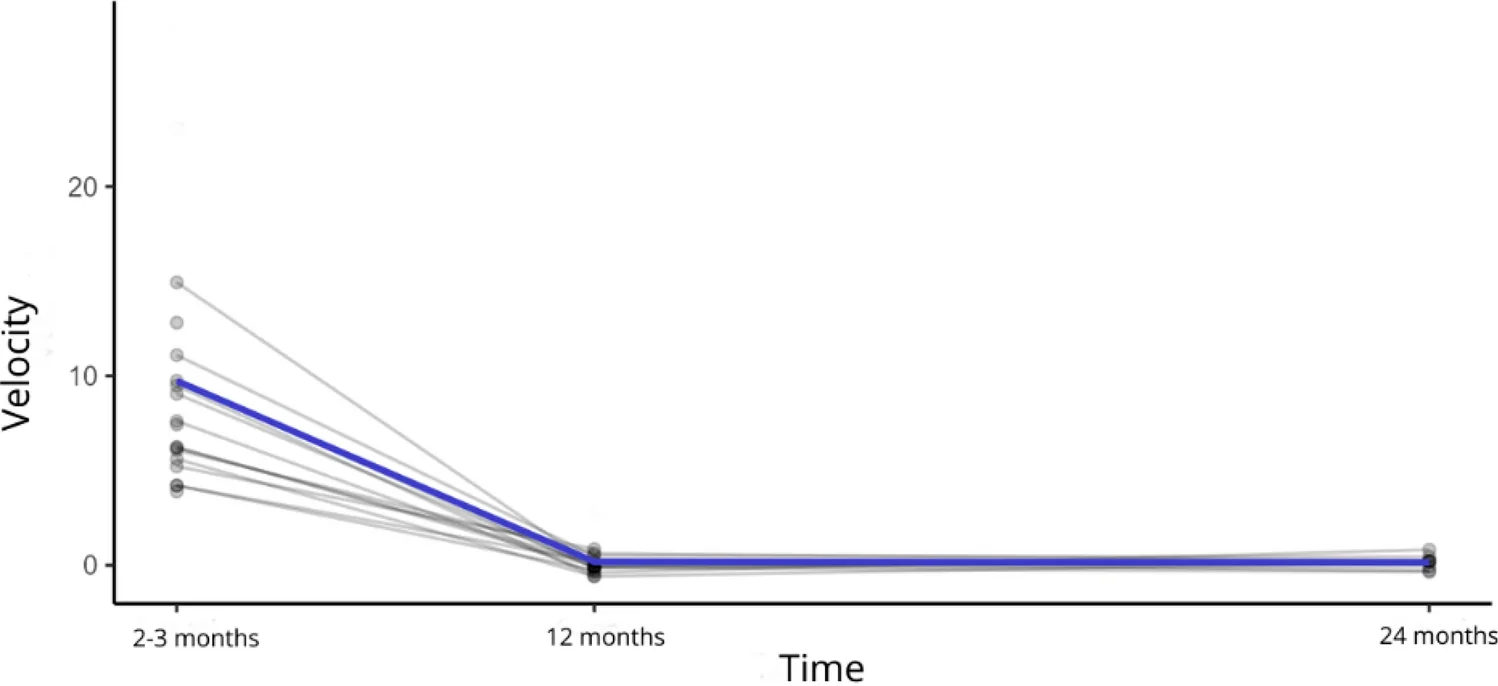
Regeneration velocity in recipients over time

For regeneration velocity (Graph 4), the variation from 2–3 months and 12 months was positively correlated with weight (p = 0.025, B = 0.17, 95%CI: [0.03, 0.30]), and postoperative CRP (p = 0.0003, B = 2.89, 95%CI: [1.66, 4.12]).

### Spleen reduction

The mean spleen reduction rate was 1.38% at 2–3 months, 11.36% at 12 months, and 10.80% at 24 months, peaking at 12 months.

#### Statistically significant correlations of the spleen reduction

**Graph 5:**
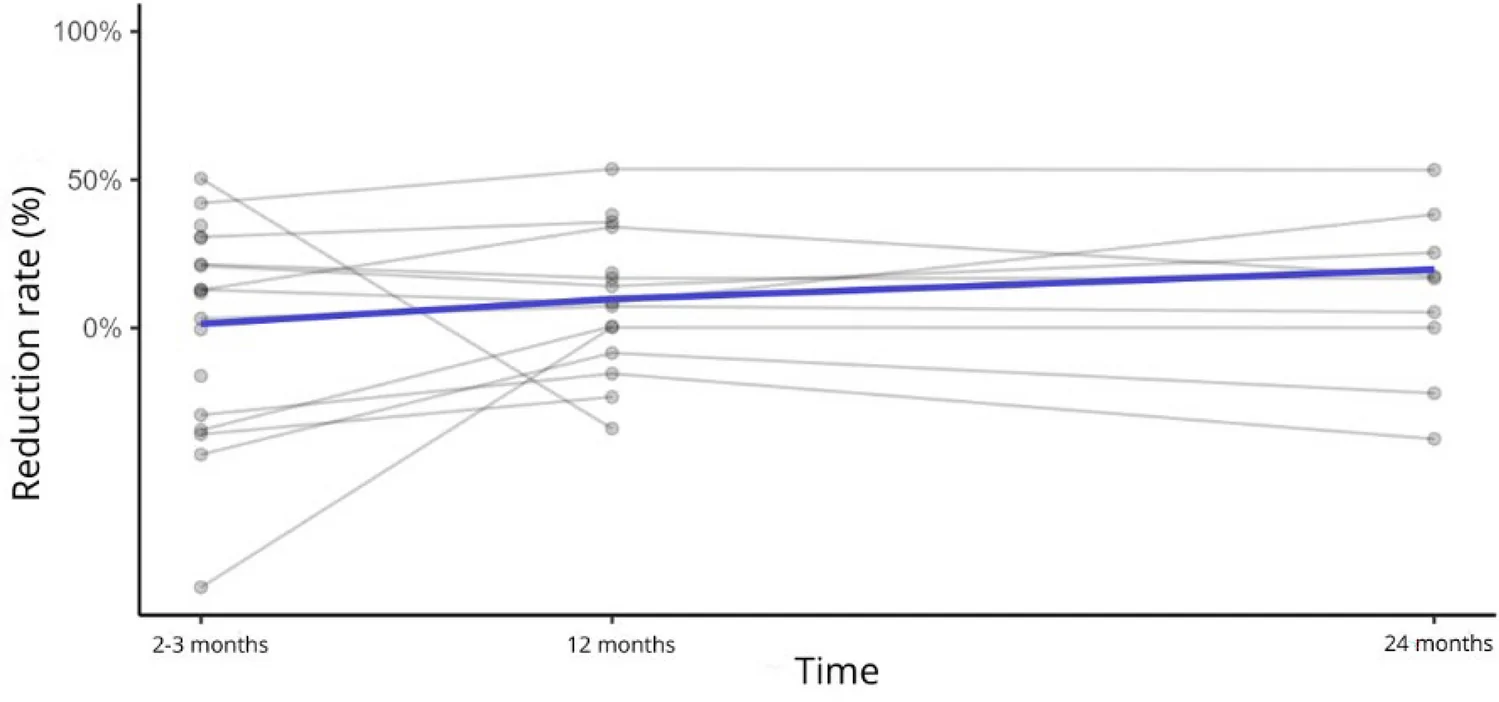
Spleen reduction rate (%) in recipients over time

The spleen reduction rate (Graph 5) over time was **negatively** correlated with hematocrit and hemoglobin at 30 days (respectively p = 0.49, B = −0.12, 95%CI: [−0.23, −0.005] and p = 0.013, B = −0.04, 95%CI: [−0.08, −0.01]). Plus, we found a **positive** relation between the variation of rate from 2–3 months and 12 months and postoperative AST and ALT (respectively p = 0.003, B = 0.08, 95%CI: [0.03, 0.13] and p = 0.005, B = 0.06, 95%CI: [0.02, 0.09]). There was also a **positive** correlation with spleen volume before transplantation (p = 0.028, B = 0.06, 95%CI: [0.01, 0.11]).

**Negative** correlation between postoperative platelets in both reduction rates at 2–3 months and 24 months (respectively p = 0.017, B = −0.19, 95%CI: [−0.33, −0.05] and (p = 0.047, B = −0.21, 95%CI: [−0.38, −0.03]) and between preoperative platelets and reduction rate at 2–3 months (p = 0.037, B = −0.13, 95%CI: [−0.24, −0.02]).

Furthermore, a **negative** correlation was found between the reduction rate at 2–3 months and postoperative hematocrit (p = 0.047, B = −0.03, 95%CI: [−0.05, −0.002]) and postoperative albumin (p = 0.016, B = −0.35, 95%CI: [−0.61, −0.10]).

No statistically significant correlation was found between liver regeneration and spleen reduction rates with the GRWR ratio.

**Graph 6:**
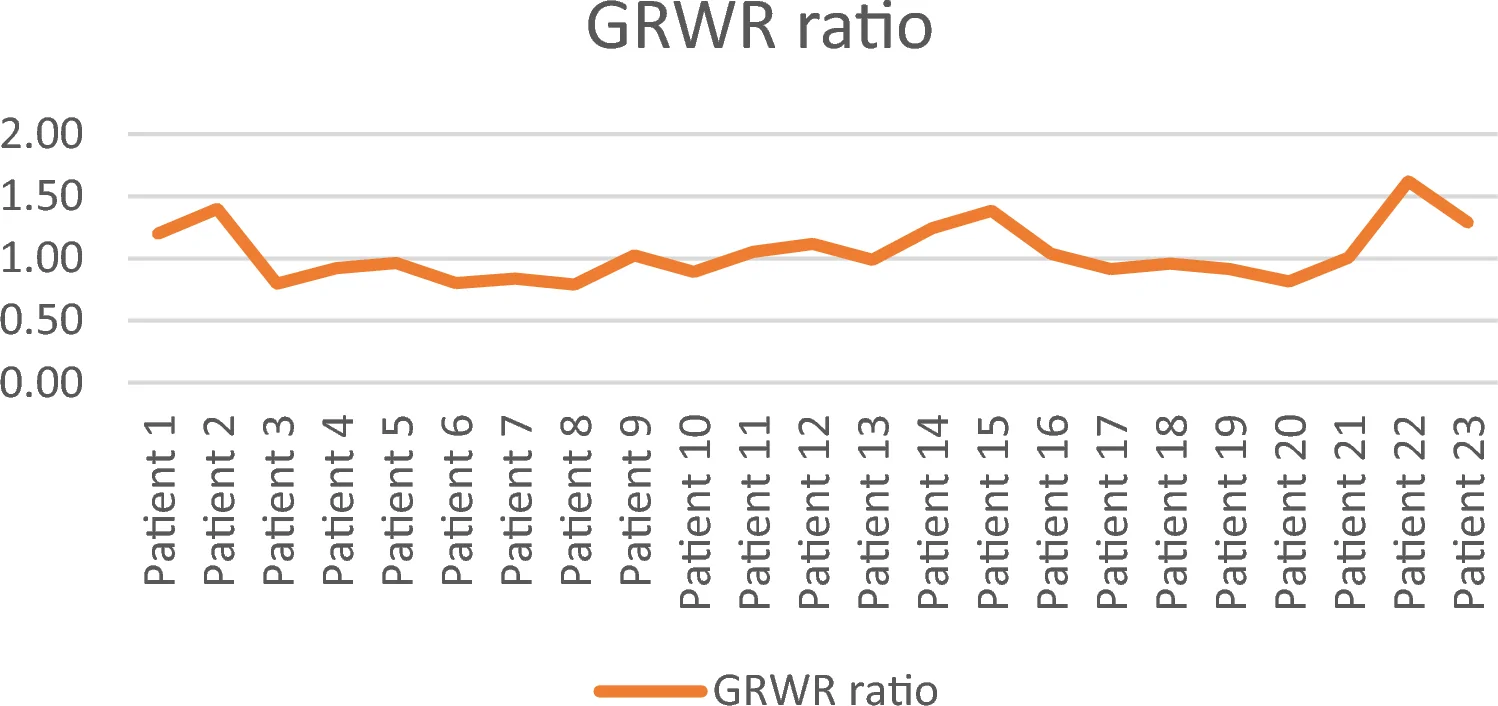
GRWR ratio

The mean GRWR ratio was 1.04, ranging between 0.79 and 1.62 (Graph 6).

### Comparison between donors and recipients

Comparing the two groups, we find, as expected, statistically significant differences in the regeneration rates, in particular at 2–3 months (p < 0.0001, B = −0.75, 95%CI: [−0.94, 0.55]) and in the variation from 2–3 months to 12 months (p = 0,005, B = −0,27, 95%CI: [−0,46, −0,09]) (Table 2). As we can see (Graph 7) the peak of regeneration is at almost 12 months in donors and already at 2–3 months for recipients, while after 12 months in both groups, the volume remains mostly stable.

**Table 2:** Mean regeneration rate at different time points in donors and recipients

RR%	Mean 2–3 m	Mean 12 m	Mean 24 m
Donors	118.70%	146.33%	154.88%
Recipients	72.61%	68.31%	79.69%

**Chart 7:**
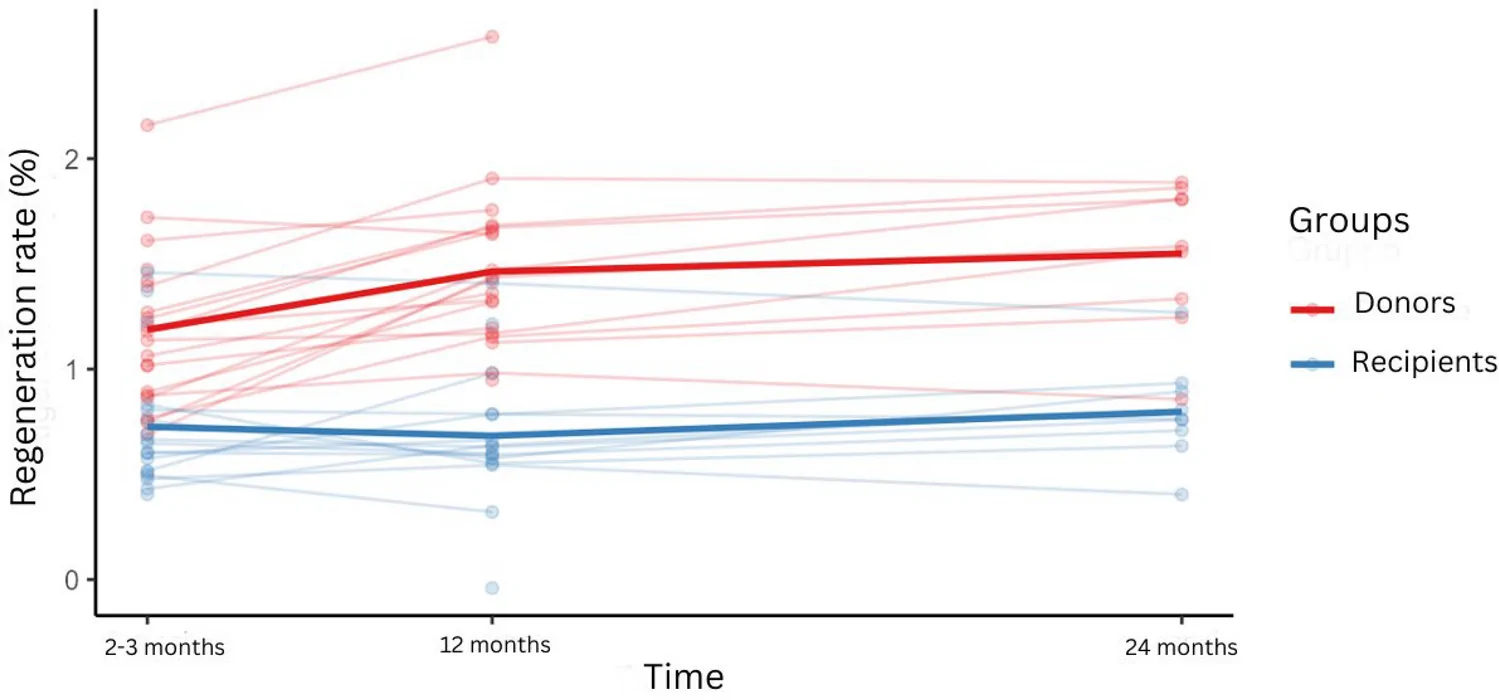
Differences between donors and recipients, in regeneration ratio in time

**Graph 8:**
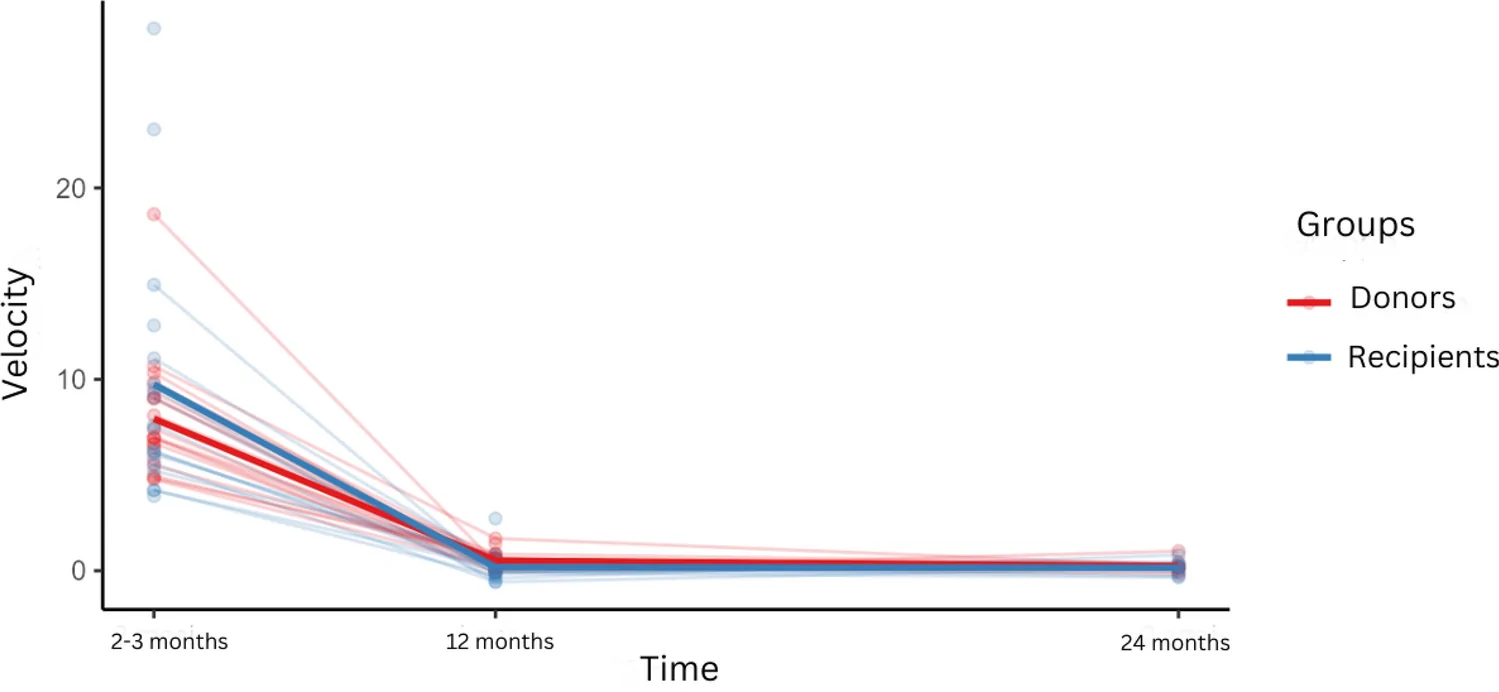
Differences between donors and recipients in regeneration velocity in time

Regarding velocity, we can say that in both groups the velocity peak is at 2–3 months, with little higher mean values in recipients, however, these differences were not statistically significant (p = 0.435, p = 0.933) (Graph 8).

Overall, all the 19 donors who have completed the 12-month follow-up have achieved over 70% hypertrophy of the initial volume before the transplant (mean 87.48, range 73.98–104.10). Meanwhile, of the 15 recipients who have completed the 12-month follow-up, 11 have reached hypertrophy greater than 70% of the estimated volume according to the Vauthey formula [39] (mean 84.2 range 51.4–123).

## Discussion

Living donor liver transplantation (LDLT) is a surgical technique that has become widespread in relation to the limited availability of organs from cadavers. It is a surgical technique that has ethical problems as the donation is performed on a healthy subject, therefore the main objective of this surgical act is to preserve the health of the donor [1, 2]*.*

The success of major liver resection in living donor liver transplantation depends on the remnant liver’s ability to regenerate. Indeed the human liver is able to regenerate because of a hyperplastic-regenerative reaction sufficient to compensate resections of 75–80% of the initial liver volume [19]. In the first analyses on the liver regeneration percentage in donors after hepatectomy, we highlighted significant differences depending on whether the right lobe, or the left lobe was resected second to standard section plane drawn about 1 cm to right of the middle hepatic vein along the Cantile imaginary line. These differences were also confirmed on the hemiliver transplanted in recipients, although less significant. In donors, separating left and right hepatectomy, there are lower regeneration values in left hepatectomies and higher rates in right hepatectomies. In recipients we found a mean regeneration rate at 2–3 months of 72.61% in right recipients and 48.92% in left recipients. We obtained the same trend in the regeneration velocity results of the right lobe vs the left lobe.

Indeed there is a negative association between regeneration rates and the residual liver volume immediately after transplantation. Specifically, smaller residual liver volumes were associated with a more robust hypertrophic response of the liver. This can be explained by the fact that reduced volume imposes a greater functional demand on the healthy residual liver, stimulating stronger regenerative processes. This also explains the much lower regeneration rates in patients who underwent left hepatectomy and that, consequently, retained the right lobe, which is volumetrically larger than the left one.

Our data confirm, as already reported in several studies [19–22], that the extension of liver resection and consequently the size of the graft or remanent liver is a major factor influencing liver regeneration.

Also Haga et al. reported in living donor liver transplantation the remaining left lobe of donors and left lobe graft of recipients regenerate more rapidly than the remaining right lobe of donors and right lobe graft of recipients [22]. Moreover, the regeneration rate is higher in the recipient’s left lobe graft than in the donor’s remaining left lobe [22].

Similarly, other authors such as Miyaoka et al. [40] reported that the volume of hepatectomy affected the mode of liver regeneration and that cellular hypertrophy significantly contributes to liver regeneration; i.e., while the remnant liver following 30% partial hepatectomy (PHx) regenerated solely by hypertrophy without cell division, hypertrophy and subsequent proliferation almost equally contributed to regeneration after 70% PHx [25, 26].

This was the reason why we concentrated subsequent analyses only on the larger, more homogeneous numerical population of right-lobe transplants, excluding the few cases of left-lobe.

As for the liver function we noticed that donors start with ALT and AST values within the reference range, which tend to increase as a typical response after resection and subsequently return to normal ranges, although stabilizing at slightly higher levels than baseline values. This suggests that the liver has recovered its functionality, and this mild enzyme elevation is an adaptive response, reflecting long-term adjustments in liver function due to the reduced volume and ongoing regenerative processes.

Another factor influencing the regenerative process is the liver function. We observed higher regeneration rates with lower preoperative ALT values; this means that elevated ALT levels which indicate preexisting liver injury or stress, could compromise the regenerative capacity of the residual liver. On the other hand, lower ALT levels may signify a healthier preoperative liver condition, providing a more favorable baseline for post-transplant regeneration and this confirms what Mohapatra et al. already reported in their study [21]. Additional corroboration of this comes from the negative association between RR% at 24 months and ALT and AST levels at 30 days, as well as between RR% at 2–3 months and postoperative bilirubin, confirming that cholestasis may impair liver regeneration, in particular cholestasis lowers portal venous flow and consequently reduces the release of hepatotropic factors [6, 16]. Thus, it can be generally concluded that liver function is a critical factor influencing the regenerative process.

The recipient group appears much more heterogeneous compared to the donors, particularly concerning the different underlying conditions that led to liver transplantation. These conditions include not only oncological diseases but also chronic inflammatory disorders. Despite this, there are various significant associations with regeneration rates, velocity regeneration, and spleen shrinkage.

Regarding the regeneration rates, we found positive correlations with spleen volume before transplantation. This reflects the complex liver-spleen interactions mediated by hemodynamic and immunological factors and could be explained by the fact that a larger spleen, which reflects portal hypertension or increased portal flow, could provide greater stimuli for liver regeneration through the release of growth factors and cytokines. At the same time, this contradicts other studies that found a negative correlation between spleen volume and liver regeneration [12, 41].

At 2–3 months, regeneration rates were significantly higher in males than in females (mean of 87% and 45.52%, respectively). These pronounced differences between genders are surprisingly exceptional, considering that authors like Shan et al. [12] have found a positive correlation between liver regeneration and female gender and knowing the role of estrogen in promoting liver regeneration and the suppression of androgen receptors, a process that occurred in both genders and that is addressed as “feminization” of the liver in males [13].

We may explain our results by saying that males usually have greater nutritional reserves and basal metabolic demands, and this may support the regeneration process. However we must consider that in the recipient group there is a disproportion between women (8) and men (15) and this can partly influence the result. Furthermore the recipient women, based on their age, should have been in menopause.

Anyhow, we think this needs further investigation before drawing any conclusions. In fact the relationships of hypertrophy with age and gender remain unclear and as reported in the work of Haga J et al. need further investigation [22].

Contrary to what we may commonly think, when it comes to blood parameters, we found that preoperative hemoglobin and hematocrit were inversely connected with RR% at 12 and 24 months. This may be explained by the fact that greater levels may imply a lower need for compensatory regeneration, considering hypoxemia as one of the factors that drive liver regeneration [6, 8]. Higher baseline values could also be associated with diseases that inhibit regeneration, such as systemic inflammation or metabolic insufficiency that could impair the regeneration process.

Furthermore, elevated Hb and Hct levels may correlate with increased iron stores, which in excessive amounts can be hepatotoxic and impair liver regeneration. Iron overload can induce oxidative stress, damaging hepatocytes and impairing regenerative signaling, and elevated hematocrit increases blood viscosity, potentially compromising microcirculation in the liver. Impaired hepatic perfusion might restrict nutrient and oxygen delivery to regenerating hepatocytes, limiting their proliferation and function. Similarly, we can explain the negative correlation between RR% at 24 months and preoperative albumin. High albumin levels indicate good nutritional status and more stable baseline liver function, which may potentially reduce the requirement for active regeneration. Low albumin levels, on the other hand, indicate that the liver was under more metabolic stress before surgery, which led to a stronger regeneration response following transplantation.

We found positive associations between RV and weight, which can be explained by the fact that heavier individuals may have higher metabolic demands, resulting in a faster regenerative response to meet homeostatic needs. Nutrition, BMI and weight are reported in the literature to be significantly associated with the regenerative process; Le Roi et al. [6] reported for example that malnutrition is associated with reduced liver regeneration after hepatectomy while Gruttadauria et al. [19] conclude that BMI is significantly associated with increased liver regeneration.

As seen in donors, a positive correlation was also discovered with postoperative CRP, an inflammation marker that can boost the regeneration process by releasing cytokines and activating the immune system. Furthermore, the favorable correlations between regeneration velocity, hemoglobin, and hematocrit at 7 days may indicate that increased oxygen levels provide metabolic support for successful regeneration.

The negative association between spleen reduction and pre- and postoperative platelet counts is particularly intriguing. Splenomegaly frequently causes thrombocytopenia due to increased platelet sequestration, which is a feature of portal hypertension. As the spleen tends to shrink following transplantation, platelet levels typically return to normal. Therefore, patients with more severe thrombocytopenia (reflecting greater splenic platelet sequestration) are more likely to experience significant spleen volume reduction as their condition improves. This emphasizes the importance of platelet improvements, a potential indicator of splenic and hepatic recovery. Also, the restoration of adequate thrombopoietin production post-liver transplantation leads to prompt restoration of platelet production [42, 43]. However, thrombocytopenia is not only related to a condition of hypersplenism but also depends on other factors such as the reduction of thrombopoietin and the reduction of platelet life span [25, 43]. In some cases, when thrombocytopenia is severe, it is associated with a delayed recovery of the platelet count after transplantation, independently of the volumetric reduction of the spleen and the type of transplant [29].

Also the positive correlation with preoperative spleen volume supports the hypothesis that larger spleens, which are frequently associated with portal hypertension, undergo more volume reduction as portal flow normalizes. Finally, the positive correlation with transaminase levels suggests that elevated AST and ALT may reflect a systemic metabolic and inflammatory response that affects not only liver regeneration but also spleen remodeling.

In recent years, the analysis of body composition parameters related to the muscle and adipose compartment has become increasingly important as it is related not only to the etiopathogenesis but also to the outcome of many chronic diseases such as oncological diseases [30, 31]. Body composition parameters extracted from CT images can be helpful in preventing chemotherapy toxicity in cancer patients and in predicting outcome or response to clinical treatments. We have learned from several works in the literature how the adipose compartment is closely related to the muscle compartment and how the presence of fat in the muscle can be indicative of poor muscle quality.

Regarding body mass composition, few are the studies, in the literature, that have evaluated the influence of body mass composition on liver regeneration. However, the negative effect of sarcopenic obesity on regeneration is well known, as demonstrated in studies such as those by Ali Deeb A. et al. and Rosenthal BE and Bittermann T. Both highlighted the positive effect of greater psoas volume (considered a measure of muscle mass) and the negative effect of higher adipose tissue levels (visceral and total) on liver regeneration, in recipients [33, 34].

Our results highlight the complex relationship between regeneration processes, muscle mass, and adipose tissue. In both donors and recipients, we did not find any correlation between RR% body mass composition parameters, but we found a significant correlation with RV.

In donors, we observed correlations between RV at 12 months and:

- SMA and SMI, positive correlation
- IMAC, negative correlation

Whereas, in recipients there were positive correlations between RV at 2–3 months and PMA, SMA, and SMI.

These results confirmed that a greater muscle mass and improved muscle quality are major stimuli to the regeneration process [33–35].

These results emphasize the importance of pretransplant body composition analysis and the early identification of sarcopenic obesity that can lead to optimizing the patient’s body composition before transplantation also considering the possibility of improving unfavorable parameters through appropriate nutritional and rehabilitative interventions.

### Donors and recipients

Our results underscore distinct patterns of liver regeneration and hypertrophy between donors and recipients, reflecting different physiological responses and underlying conditions in each group, due to the heterogeneity of the two groups, primarily since donors are healthy individuals, whereas recipients often present multiple underlying conditions in addition to the liver disease that led to the transplant. That said, as expected, **the regeneration rates were higher in donors compared to recipients**, with statistically significant differences, also considering that donors start with smaller residual volumes than recipients, as they retain the left liver lobe, which theoretically represents about 40% of the total liver volume.

Moreover, we found that recipients have a growth peak within 2–3 months, while donors experienced more progressive growth that peaks at 12 months. At 2–3 months, we found that recipients had somewhat higher velocities than donors, though these differences were not statistically significant. The donor and recipient shared the same normal liver parenchyma but the regeneration is faster in recipients than in donors. We may therefore conclude that the physiological urgency to restore liver function after transplantation, driven by metabolic demands and systemic adaptations, is likely reflected in the accelerated early regeneration in recipients, furthermore, the immediate regeneration in recipients may be related to high liver blood flow due to persistence of a hyperdynamic state, immunosuppressant administration, or humoral factors [22].

In contrast, the regenerative response in donors is more gradual, indicating a less urgent but steady recovery of liver volume after the hepatectomy. The liver volumes stabilize in both groups, after 12 months, suggesting that the regenerative processes have mostly reached a plateau. This stabilization implies that, in relation to the host's metabolic and systemic requirements, the liver has attained a functioning balance by this point.

Another factor that confirms the different regeneration patterns is the percentage of liver recovery relative to the initial total liver volume in donors and the estimated liver volume, calculated according to Vauthey's formula, in recipients. We can observe that at time 0, the percentages are higher in recipients because they start with a bigger volume, the right lobe. Subsequently, there is a rapid increase in both, with a peak for recipients followed by a slight decline between 3 and 12 months, while donors show a progressively slow increase. As postulated by Haga et al. [22], donors do not recover 100% of the initial volume, and recipients do not achieve 100% of the estimated volume, although the final normal liver function. Livers did not return to their full volume neither in donors nor in recipients but still functioned well without graft failure and with normal liver function: this suggests that livers can function well without returning to the initial volume [22].

## Limits

Our study has some limitations. The first is the small sample size, which is justified by the highly specialized nature of the study. The donor group, consisting of healthy subjects, is homogeneous, while the recipient group is heterogeneous given the different etiologies included. In light of the above, our results can be considered preliminary, partly consistent with other studies in the literature and partly a starting point for new, more numerically robust studies.

Another limitation is the lack of a standardized protocol for recipient follow-up after transplantation, as instrumental tests in this population are generally based on clinical needs, while standardizing follow-up in the donor group is easier. This limitation resulted in a lack of follow-up data, particularly for recipients.

Despite these limitations, the strength of this study is having compared the liver regeneration process in donors and recipients, analyzing the different modalities and considering the main variables in significant association.

## Conclusion

We can conclude that the liver regeneration process is influenced by numerous factors, which are easier to identify in donors because of their homogeneity as a group.

Specifically, in donors, the residual liver volume after transplantation and pre-transplant liver function profile plays a significant role, and both are negatively correlated with regeneration.

Our work also confirms the close relationships between liver regeneration and body composition in both donors and recipients, further underlining the systemic relationships of the fascinating liver regenerative process.

In recipients negative correlations with hematological and nutritional parameters were seen,. Moreover, the significant interplay between the liver and spleen is highlighted, in particular, liver regeneration is a process that goes parallel with the volumetric reduction of the spleen, both influenced by the recipient's systemic condition and by local hepato-splenic factors.

Overall although the liver parenchymal structure is the same in donors and recipients, the regeneration processes in donors and recipients happen differently and at various times, with donors showing a higher capacity for regeneration and a better ability to withstand the stress of surgery.

In conclusion our study emphasizes how sophisticated and versatile the process of liver regeneration is, influenced not only by the remaining liver or graft, but also or above all by the metabolic environment of the host; it confirms existing knowledge about liver regenerative capacity, the importance of liver volumetric analysis in planning living-donor liver transplantation, and explores the relationship between liver regeneration and systemic metabolic conditions, as well as local hepatosplenic factors.

## Supplementary Information

Below is the link to the electronic supplementary material.

Supplementary file1 (PPTX 41 KB)

Supplementary file2 (DOCX 44 KB)

## Data Availability

The data collected to support the results discussed in the work are available in anonymous excel database format if the editorial board deems them necessary.
