# Supplementary material for: Liver regeneration after living donor liver transplantation (LDLT)
Source: Abdom Radiol (NY). 2026 Jun 5;51(12):6529–44. doi: 10.1007/s00261-026-05567-1 (PMC13768376; doi:10.1007/s00261-026-05567-1)
Supplement: Supplementary file 1 [file 261_2026_5567_MOESM2_ESM.docx]

**SUPPLEMENTARY FILE**

**Table 1**

| **Variable** | **N** | **Missing** | **Mean** | **Median** | **SD** | **Minimum** | **Maximum** |
| --- | --- | --- | --- | --- | --- | --- | --- |
| PLATELETS | 23 | 0 | 240.565 | 235 | 66.40 | 145 | 377 |
| AST | 23 | 0 | 19.783 | 19 | 5.94 | 13 | 39 |
| ALT | 23 | 0 | 21.391 | 21 | 8.66 | 7 | 40 |
| GGT | 21 | 2 | 22.857 | 17 | 14.39 | 12 | 67 |
| TOT BIL. | 23 | 0 | 0.556 | 0.490 | 0.30 | 0.3 | 1.640 |
| DIR BIL. | 20 | 3 | 0.305 | 0.300 | 0.02 | 0.3 | 0.4 |
| PCR | 21 | 2 | 0.224 | 0.200 | 0.09 | 0.2 | 0.6 |

Table 1. Donors: preoperative bio-humoral values. SD = Standard deviation

**Table 2**

| **Variable** | **N** | **Missing** | **Mean** | **Median** | **SD** | **Minimum** | **Maximum** |
| --- | --- | --- | --- | --- | --- | --- | --- |
| PLATELETS | 23 | 0 | 236.00 | 216 | 78.04 | 130 | 401 |
| AST | 23 | 0 | 384.52 | 387 | 129.20 | 184 | 745 |
| ALT | 23 | 0 | 401.26 | 367 | 132.70 | 168 | 676 |
| TOT BIL. | 23 | 0 | 2.16 | 1.97 | 0.88 | 1.12 | 4.58 |

Table 2. Donors: postoperative bio-humoral values. SD = Standard deviation

**Table 3**

| **Variable** | **N** | **Missing** | **Mean** | **Median** | **SD** | **Minimum** | **Maximum** |
| --- | --- | --- | --- | --- | --- | --- | --- |
| PLATELETS | 23 | 0 | 253.652 | 240 | 77.13 | 115 | 498 |
| AST | 23 | 0 | 84.304 | 60 | 54.93 | 30 | 223 |
| ALT | 23 | 0 | 153.957 | 126 | 79.07 | 18 | 357 |
| GGT | 23 | 0 | 264.522 | 136 | 287.84 | 17 | 1307 |
| TOT BIL. | 23 | 0 | 1.834 | 1.690 | 1.16 | 0.450 | 5.06 |
| DIR BIL. | 21 | 2 | 0.793 | 0.540 | 0.70 | 0.300 | 3.15 |
| PCR | 23 | 0 | 1.552 | 1.300 | 1.17 | 0.200 | 4.20 |

Table 3. Donors:bio-humoral values 7 days postoperative. SD = Standard deviation

**Table 4**

| **Variable** | **N** | **Missing** | **Mean** | **Median** | **SD** | **Minimum** | **Maximum** |
| --- | --- | --- | --- | --- | --- | --- | --- |
| PLATELETS | 22 | 1 | 273.591 | 260.50 | 79.08 | 176 | 523 |
| AST | 22 | 1 | 35.955 | 30.00 | 23.65 | 16 | 130 |
| ALT | 22 | 1 | 41.227 | 37.50 | 27.79 | 11 | 119 |
| GGT | 22 | 1 | 200.273 | 147.00 | 194.72 | 16 | 862 |
| TOT BIL. | 22 | 1 | 0.666 | 0.635 | 0.23 | 0.350 | 1.300 |
| DIR BIL. | 22 | 1 | 0.310 | 0.300 | 0.03 | 0.300 | 0.460 |
| PCR | 22 | 1 | 0.873 | 0.650 | 0.55 | 0.200 | 1.900 |

Table 4. Donors: bio-humoral values 30 days postoperative. SD = Standard deviation

**Table 5**

| **Body composition** | **Variable** | **Mean** | **Median** | **SD** | **Minimum** | | **Maximum** | |
| --- | --- | --- | --- | --- | --- | --- | --- | --- |
| Muscle mass | PMA | 17.609 | 17 | 5.17 | 8 | | 29 | |
|  | SMA | 129.435 | 129 | 28.36 | 89 | | 190 | |
|  | SMI | 46.200 | 45.650 | 7.77 | 33.500 | | 60.650 | |
|  | MA | 56.913 | 57 | 4.64 | 42 | | 64 | |
|  | IMAC | -0.520 | -0.516 | 0.07 | -0.732 | | 0.365 | |
| Adipose tissue | HU adip. sub. tissue | -110.435 | -112 | 7.982 | | -119 | -82 |  |
|  | VFA | 84.060 | 80.470 | 51.414 | | 9.500 | 193.49 |  |
|  | SFA | 147.428 | 120.020 | 86.104 | | 19.240 | 341.52 |  |
|  | VFI | 29.732 | 27.750 | 16.964 | | 3.580 | 62.43 |  |
|  | SFI | 53.191 | 45.170 | 30.812 | | 6.140 | 121.73 |  |
|  | TFI | 78.301 | 81.610 | 39.643 | | 11.050 | 161.17 |  |
|  | VSR | 0.702 | 0.450 | 0.539 | | 0.210 | 2.28 |  |

Table 5. Donors: body composition parameters. SD = Standard deviation

**Tab 7**

| **Variable** | **N** | **Missing** | **Mean** | **Median** | **SD** | **Minimum** | **Maximum** |
| --- | --- | --- | --- | --- | --- | --- | --- |
| Hb | 23 | 0 | 12.530 | 12.900 | 1.806 | 8.300 | 15.40 |
| Hct | 23 | 0 | 38.496 | 39.400 | 5.301 | 26.500 | 47.10 |
| PLATELETS | 23 | 0 | 153.391 | 113 | 144.139 | 38 | 599 |
| AST | 23 | 0 | 52.609 | 44 | 29.808 | 20 | 139 |
| ALT | 23 | 0 | 48.739 | 34 | 43.835 | 15 | 215 |
| GGT | 16 | 7 | 268.250 | 113.500 | 485.226 | 21 | 1926 |
| TOT BIL. | 23 | 0 | 2.080 | 1.120 | 3.065 | 0.370 | 15.19 |
| DIR BIL. | 14 | 9 | 1.478 | 0.300 | 3.601 | 0.300 | 13.88 |
| ALBUMIN | 23 | 0 | 3.691 | 3.800 | 0.619 | 2.300 | 4.80 |
| PCR | 22 | 1 | 0.714 | 0.400 | 0.922 | 0.200 | 4.40 |

Table 7. Recipients: preoperative bio-humeral values. SD = Standard Deviation.

**Tab 8**

| **Variable** | **N** | **Missing** | **Mean** | **Median** | **SD** | **Minimum** | **Maximum** |
| --- | --- | --- | --- | --- | --- | --- | --- |
| Hb | 23 | 0 | 10.33 | 9.700 | 1.763 | 8.100 | 14.40 |
| Hct | 23 | 0 | 30.72 | 28.500 | 5.808 | 23.500 | 44.70 |
| PLATELETS | 23 | 0 | 127.43 | 76 | 114.684 | 26 | 416 |
| AST | 22 | 1 | 337.09 | 301.00 | 296.749 | 12 | 1305 |
| ALT | 23 | 0 | 421.78 | 288 | 445.314 | 14 | 1704 |
| TOT BIL. | 23 | 0 | 3.45 | 2.910 | 2.628 | 0.460 | 12.05 |
| ALBUMIN | 23 | 0 | 3.00 | 2.800 | 0.524 | 2.500 | 4.70 |
| PCR | 21 | 2 | 1.42 | 0.900 | 1.317 | 0.200 | 4.30 |

Table 8. Recipients: postoperative bio-humeral values. SD = Standard Deviation

**Tab 9**

| **Variable** | **N** | **Missing** | **Mean** | **Median** | **SD** | **Minimum** | **Maximum** |
| --- | --- | --- | --- | --- | --- | --- | --- |
| Hb | 23 | 0 | 9.94 | 9.700 | 1.405 | 7.300 | 12.80 |
| Hct | 23 | 0 | 29.81 | 29.500 | 4.113 | 21.700 | 38.00 |
| PLATELETS | 23 | 0 | 129.35 | 101 | 91.566 | 34 | 444 |
| AST | 23 | 0 | 91.43 | 77 | 54.262 | 28 | 210 |
| ALT | 23 | 0 | 265.61 | 231 | 199.082 | 51 | 932 |
| GGT | 23 | 0 | 231.91 | 180 | 214.358 | 40 | 846 |
| TOT BIL. | 23 | 0 | 2.35 | 1.880 | 1.353 | 1.040 | 6.62 |
| DIR BIL. | 21 | 2 | 1.26 | 0.900 | 1.195 | 0.300 | 4.70 |
| ALBUMIN | 23 | 0 | 3.57 | 3.400 | 0.500 | 2.600 | 4.70 |
| PCR | 23 | 0 | 1.41 | 0.900 | 1.378 | 0.200 | 6.10 |

Table 9. Recipients: bio-humeral values 7 days postoperative. SD = Standard Deviation.

**Tab 10**

| **Variable** | **N** | **Missing** | **Mean** | **Median** | **SD** | **Minimum** | **Maximum** |
| --- | --- | --- | --- | --- | --- | --- | --- |
| Hb | 23 | 0 | 10.904 | 11.000 | 1.401 | 7.800 | 13.50 |
| Hct | 23 | 0 | 34.409 | 34.800 | 4.602 | 24.100 | 41.80 |
| PLATELETS | 23 | 0 | 221.522 | 229 | 103.359 | 64 | 492 |
| AST | 23 | 0 | 29.652 | 21 | 23.866 | 10 | 119 |
| ALT | 23 | 0 | 53.000 | 40 | 40.794 | 12 | 175 |
| GGT | 23 | 0 | 170.609 | 69 | 279.785 | 15 | 1243 |
| TOT BIL. | 23 | 0 | 1.127 | 0.690 | 1.371 | 0.360 | 5.46 |
| DIR BIL. | 23 | 0 | 0.515 | 0.300 | 0.563 | 0.300 | 2.77 |
| ALBUMIN | 23 | 0 | 3.613 | 3.700 | 0.365 | 2.700 | 4.10 |
| PCR | 23 | 0 | 3.239 | 1.300 | 4.496 | 0.200 | 17.60 |

Table 10. Recipients: bio-humeral values 30 days postoperative. SD = Standard Deviation.

**Tab 11**

| **Body composition** | **Variable** | **Mean** | **Median** | **SD** | **Minimum** | | **Maximum** | |
| --- | --- | --- | --- | --- | --- | --- | --- | --- |
| Muscle mass | PMA | 15.628 | 15.140 | 4.41 | 7.860 | | 24.160 | |
|  | SMA | 127.721 | 131.630 | 27.94 | 80.550 | | 209.770 | |
|  | SMI | 45.650 | 46.450 | 8.23 | 28.880 | | 66.210 | |
|  | MA | 52.174 | 52 | 6.39 | 37 | | 63 | |
|  | IMAC | -0.496 | -0.472 | 0.09 | -0.708 | | -0,349 | |
| Adipose tissue | HU adip. sub. tissue | -106.435 | -108 | 8.95 | | -116 | -82 |  |
|  | VFA | 139.108 | 106.210 | 69.00 | | 58.250 | 283.310 |  |
|  | SFA | 162.289 | 166.340 | 70.33 | | 53.530 | 315.360 |  |
|  | VFI | 49.742 | 39.550 | 24.19 | | 19.690 | 97.330 |  |
|  | SFI | 59.169 | 64.220 | 27.61 | | 17.280 | 123.190 |  |
|  | TFI | 108.911 | 110.780 | 44.51 | | 46.560 | 214.580 |  |
|  | VSR | 0.952 | 0.910 | 0.43 | | 0.310 | 1.690 |  |

Table 11: Recipients’ body composition parameters. SD=Standard deviation

**Tab 12**

**Table 12: mean values of regeneration rate and velocity in donors and recipients in the indicated time intervals.**
